# Supplementary material for: Visual field differences in temporal synchrony processing for audio-visual stimuli
Source: PLoS One. 2021 Dec 16;16(12):e0261129. doi: 10.1371/journal.pone.0261129 (PMC8675747; doi:10.1371/journal.pone.0261129)
Supplement: Attachment — Submitted filename: Reply_PO3.docx [file pone.0261129.s001.docx]

Replies to Editor’s comments

I apologize that the previous manuscript did not meet the PLOS ONE style format. I have revised the format according to the following comments.

1. Please ensure that your manuscript meets PLOS ONE's style requirements, including those for file naming. The PLOS ONE style templates can be found at

https://journals.plos.org/plosone/s/file?id=wjVg/PLOSOne_formatting_sample_main_body.pdf and

https://journals.plos.org/plosone/s/file?id=ba62/PLOSOne_formatting_sample_title_authors_affiliations.pdf

*Reply:* I have revised the manuscript format according to PLOS ONE’s style requirements.

2. In line with PLOS' guidelines on detailed reporting (https://journals.plos.org/plosone/s/criteria-for-publication#loc-3), please ensure that you have provided sufficient detail on participant recruitment in the Methods section, including from where participants were recruited.

*Reply:* I have added descriptions about the details on participant recruitment in each experiment section.

3. Your ethics statement should only appear in the Methods section of your manuscript. If your ethics statement is written in any section besides the Methods, please move it to the Methods section and delete it from any other section. Please ensure that your ethics statement is included in your manuscript, as the ethics statement entered into the online submission form will not be published alongside your manuscript.

*Reply:* I have moved the descriptions of the ethics statement from the Participants section to the independent Ethics statement section.

Replies to Reviewer 1’s comments

I appreciate your helpful and valuable comments on this manuscript. I have revised the manuscript to clarify the theoretical motivation and rationale interpretation of this study.

*Point 1:* Page 2, Lines 11-13

"...exhibited more auditory preceding timing in the peripheral....."

Not clear what this means. Please rephrase.

*Reply:* I have rephrased this description to clarify the meaning of the text (page 2, lines 22-25).

“The results indicate that auditory stimuli should be presented earlier for visual stimuli in the central visual field than in the peripheral visual field condition for one to perceive subjective simultaneity in the temporal order judgment task conducted in this study.”

*Point 2:* "On the other hand...." Other hand relative to what? Consider deleting this cliche

*Reply:* I have deleted the pertinent text.

*Point 3:* “temporal binding window was larger…”
Presumably, this refers to a broader subjective simultaneity bandwidth, not amplitude. Please specify.

*Reply:* I revised this statement as per your suggestion (page 2, lines 25-27).

“Meanwhile, the subjective simultaneity bandwidth was broader in the central visual field than in the peripheral visual field during the simultaneity judgment task.”

*Point 4:* Line 21-23
I don't follow this. Which visual features are the authors referring to? Temporal response? If so, what kind of temporal response exactly?

*Reply:* The purpose of this study was to investigate the effects of differences in visual processing speed (including temporal resolution) based on visual fields on temporal synchrony perception of audio-visual stimuli. I have revised the passage to clarify the purpose (page 2, lines 31-35).

“These results suggest that differences in visual processing speed based on the visual field modulate the temporal processing of audio-visual stimuli. Future studies are necessary to confirm the effects of compensation regarding differences in the temporal resolution of the visual filed in later cortical visual pathway on visual field differences in audio-visual temporal synchrony.”

*Point 5:* Page 4 Line 18-23
The authors need to explain for the reader why they are comparing simultaneity judgment with TOJ tasks. The paragraph (lines 18-23) doesn't provide a justification.

*Reply:* I have added explanations as to why SJ was compared with TOJ tasks (page 5, lines 86-89).

“Van Eijk, Kohlrausch, Juola, and van de Par [26] have shown a lack of correlation between SJ and TOJ PSS, and proposed a different kind of sensitivity between TOJ and SJ for audio-visual asynchrony. Therefore, it is necessary to use both TOJ and SJ tasks to examine audio-visual temporal synchrony.”

*Point 6:* Line 22
‘view distances…”
Presumably, the authors are referring to variations in eccentricity arising from variations in viewing distance of an otherwise identical stimulus. This needs to be clarified.

*Reply:* In Kopinska and Harris’s study, eccentricity and viewing distance were manipulated individually. I have revised the descriptions to clarify this point (page 4, line 80-page 5, line 83).

“Kopinska and Harris [11] compared the PSS between central (0° eccentricity) and peripheral (20° eccentricity) VFs using the TOJ task by manipulating both the eccentricity of the visual stimulus and participants’ viewing distance and showed that the timing of subjective simultaneity did not differ between VFs.”

*Point 7:* Page 5 Line 7
It's not clear from the Introduction what the theoretical motivation for the visual eccentricity (visual field) manipulation is.

*Reply:* My previous study reported that the difference in visual processing speed based on spatial frequency modulates audio-visual temporal synchrony perception by using the SJ task. Therefore, this study was conducted to confirm this finding by manipulating the eccentricity of visual stimuli, which is also associated with visual processing speed. I have added the descriptions about this theoretical motivation (page 4, lines 64-78).

“Temporal synchrony perception for audio-visual stimuli is affected by the processing speed of the visual system. Previous studies have reported that the PSS for audio-visual stimuli was directed to more auditory leads in low spatial frequency stimuli than in high spatial frequency stimuli [20, 21]. Visual systems have two channels, namely the transient and sustained channels, with each kind having different temporal resolutions (processing speeds) [22]. Transient channels respond to low spatial frequencies and exhibit high temporal resolution, whereas sustained channels exhibit high spatial frequencies and low temporal resolution [23]. Therefore, auditory stimuli should be presented earlier for low spatial frequency stimuli than for high spatial frequency stimuli for one to perceive subjective simultaneity. These two channels are projected from different retinal positions [24]. The transient channel is projected from the peripheral visual field (VF), while the sustained channel is projected from the central VF; thus, the processing speed of visual stimuli in the central VF is slower than that in the peripheral VF. The present study confirmed the effects of visual temporal resolution on audio-visual synchrony perception by manipulating the VF in which the visual stimulus is presented (i.e., eccentricity). “

*Point 8:* There are no obvious hypotheses pertaining to this manipulation for any of the three tasks or for the effects of temporal recalibration

*Reply:* I did not set obvious hypotheses for any of the three tasks since the purpose of this study was to confirm the differences in PSS due to the visual processing speed. For rapid recalibration, I added some predictions in the revised manuscript. (page 5, line 102-page 6, line 108).

“Using the SJ task, Takeshima [30] reported that normal rapid temporal recalibration occurred regardless of a difference in visual processing speed based on spatial frequency. The present study predicts that normal rapid temporal recalibration would be observed in both the central and peripheral VFs in the SJ task. In the TOJ task, as in Roseboom [29], temporal recalibration in the direction opposite to that in the SJ task is predicted to occur. This study also explores rapid temporal recalibration using SB perception since previous studies have not examined this using the paradigm under investigation. “

*Point 9:* Page 10, Lines 19-21
Reference to neural transmission speed is an interpretation, and possibly a prediction/hypothesis. Either way, this doesn't belong in the Results section.

“…a vision leads response is suppressed…’ [italics added]
Again, this is an interpretation, not a description of the experimental Results

Page 14, Lines 6-7
Again, this is an interpretation, not a description of the experimental Results

*Reply:* In the revised manuscript, I have clearly divided the “Results and discussion” section of the previous manuscript into a “Results” section and a “Discussion” section in each experiment.

*Point 10:* Page 20, Lines 12-13
How do you know they are more suppressed? It is more descriptive to say that TOJ vision leads responses are less frequent in the visual periphery than in central vision. To invoke suppression implies a mechanism for which these experiments offer no evidence

*Reply:* Although the result of the TOJ task was inconsistent with the VF difference in temporal resolution, it was consistent with that in visual latency in previous studies. I have discussed this point in the revised manuscript (page 12, line 245-255).

“The difference observed between the central and peripheral VFs was opposite to that predicted by the difference in temporal resolution. The temporal resolution is higher for the peripheral VF than for the central VF [24]; thus, it was predicted that auditory stimuli should be presented earlier for visual stimuli in the peripheral VF than in the central VF condition for one to perceive subjective simultaneity. However, the current observed difference in the PSS was consistent with the difference in visual latency between the central and peripheral VFs. The response time for the visual stimulus was shorter at the central VF than at the peripheral VF [35]. A previous study that manipulae spatial frequency also observed differences in the PSS that were consistent with differences observed in response time [20, 30]. Therefore, in a TOJ task, differences in the PSS between central and peripheral VFs could be attributed to differences in the response time, not temporal resolution.”

*Point 11:* Page 20, Lines 12-13
How do you know they are more suppressed? It is more descriptive to say that TOJ vision leads responses are less frequent in the visual periphery than in central vision. To invoke suppression implies a mechanism for which these experiments offer no evidence

*Reply:* I have revised the implications for the description of the PSS value (page 23, line 512-513).

“Experiment 1 showed that the PSS value was smaller in the central VF condition than in the peripheral VF condition.”

*Point 12:* Lines 14-15
Presumably, the authors are referring to the observation that SJ derived TBWs are broader at central than at peripheral visual field locations.

*Reply:* I have revised the sentence to refer to the VF difference in TBW width (page 23, lines 514-515).

“Experiment 2 indicated that the TBW was broader at the central VF than at the peripheral VF.”

*Point 13:* Page 22, Lines 22-23
I don't follow this sentence. Which temporal characteristics exactly are the authors referring to?

*Reply:* I have revised the last paragraph of the General discussion section to clarify the claim of this study (page 26, lines 561-567).

“The present findings also suggest a discrepancy in judgment between the VFs; judging simultaneity between visual events and sound in the central VF, whereas judging bounce induced by sound in the peripheral VFs. The use of SB perception in investigating temporal synchrony perception for audio-visual stimuli needs to be carefully considered. Moreover, bounce responses differ between the VFs (i.e., the results of a no-sound experiment and alpha values). It is necessary to further investigate whether this difference affects the estimation of the PSS and TBW of SB perception.”

*Point 14:* Nor is it clear how the temporal characteristics of the visual field ought to explain the diverse pattern of task dependent visual field effects reported here.

*Reply:* Thank you for your comment. Based on this, I have reinterpreted the results regarding the VF difference in this study (page 24, line 523-page 25, line 539).

“VF differences in audio-visual temporal synchrony were observed in the TOJ and SJ tasks. In a TOJ task, the auditory stimulus is presented earlier as a visual stimulus for one to perceive subjective simultaneity in the central than in the peripheral VF condition. In an SJ task, the TBW width of the central VF is broader than that of the peripheral VF. The difference in the PSS of the TOJ task was consistent with the VF difference in a visual latency, whereas the difference in the TBW of the SJ task was consistent with the VF difference in a temporal resolution. As a preliminary prediction, the VF differences in the PSS and TBW both follow differences in temporal resolution. It has been shown that the difference in eccentricity-dependent temporal resolution are observed in the early visual cortex and are compensated later in the cortical visual pathway [39]. A TOJ task is assumed to be associated with higher-order processing compared with an SJ task [26, 40]. Therefore, the discrepancy between VF difference in the PSS and temporal resolution could be attributed to the visual hierarchy of eccentricity-dependent temporal contrast in a TOJ task. Moreover, a TOJ task has been proposed to be sensitive to PSS change [26], whereas an SJ task has been proposed to be sensitive to TBW change [41]. Thus, the discrepancy in the indices for which VF differences were observed between the TOJ and SJ tasks would reflect the difference in sensitivity of indices to audio-visual synchrony perception.”

Replies to Reviewer 2’s comments

I appreciate your helpful and valuable comments on this manuscript and have revised it based on your comments.

Major comments:

1. In order to better predict and explain the results observed in the TOJ, SJ, and SB paradigms, the critical differences of their underlying mechanisms should be introduced and discussed. This is especially critical when different results were observed in each paradigm: should these differences be attributed to the different sensory, perceptual, or decisional processing?
As the author mentioned in the Discussion, SJ and TOJ may share the same perceptual mechanism but different decisional processes (e.g., García-Pérez & Alcalá-Quintana, 2015). On the other hand, the SB perception may involve causal relations and attention in addition to audiovisual integration (Shimojo et al., 2001; van Eijk et al., 2008). Hence, stronger theoretical backgrounds will be necessary to understand the distinct results observed in three experiments, and will provide a clearer rationale for the current study.

*Reply:* I have reinterpreted the results of the VF difference between the TOJ and SJ tasks based on the compensation of the difference in temporal resolution of the visual filed in later cortical visual pathway (page 24, line 525-page 25, line 539).

“VF differences in audio-visual temporal synchrony were observed in the TOJ and SJ tasks. In a TOJ task, the auditory stimulus is presented earlier as a visual stimulus for one to perceive subjective simultaneity in the central than in the peripheral VF condition. In an SJ task, the TBW width of the central VF is broader than that of the peripheral VF. The difference in the PSS of the TOJ task was consistent with the VF difference in a visual latency, whereas the difference in the TBW of the SJ task was consistent with the VF difference in a temporal resolution. As a preliminary prediction, the VF differences in the PSS and TBW both follow differences in temporal resolution. It has been shown that the difference in eccentricity-dependent temporal resolution are observed in the early visual cortex and are compensated later in the cortical visual pathway [39]. A TOJ task is assumed to be associated with higher-order processing compared with an SJ task [26, 40]. Therefore, the discrepancy between VF difference in the PSS and temporal resolution could be attributed to the visual hierarchy of eccentricity-dependent temporal contrast in a TOJ task. Moreover, a TOJ task has been proposed to be sensitive to PSS change [26], whereas an SJ task has been proposed to be sensitive to TBW change [41]. Thus, the discrepancy in the indices for which VF differences were observed between the TOJ and SJ tasks would reflect the difference in sensitivity of indices to audio-visual synchrony perception.”

2. The author proposed an interesting assumption that visual stimulus presented to sustained channel and transient channel may lead to different audiovisual simultaneity perception, because the transient channel has higher temporal resolution and faster processing speed. However, these two characteristics in the temporal processing may be associated with different aspects of audiovisual simultaneity perception. In my intuitive guessing, higher temporal resolution may be associated with narrower audiovisual TBW, whereas the faster processing time may be associated with the shift of PSS (see the model of García-Pérez & Alcalá-Quintana, 2012, and a recent study by Chien et al., 2020). I also found that García-Pérez and Alcalá-Quintana published a paper last year (2020) on this issue.

Hence, I would expect that the PSS should be at more negative (i.e., the auditory stimulus should be presented earlier) in the periphery than in the center, if visual processing is faster in the periphery than in the center. This prediction was not consistent with the results, and even an opposite direction was observed in Experiment 1. I cannot follow the author’s “suppression” account on p. 10.

On the other hand, the result of TBW in Experiment 2 was consistent with my prediction that the TBW was narrower in the periphery than in the center because of the higher temporal resolution in the periphery. However, this result contrasts with trend as a function of eccentricity reported by Stevenson et al. (2012). Please discuss this critical inconsistency.

*Reply:* Visual latency is shorter in the central VF than in the peripheral VF, whereas temporal resolution is higher in the peripheral VF than in the central VF. The PSS result in the TOJ task was consistent with the prediction because of this visual latency difference. Thus, I have reinterpreted the PSS results in Experiment 1 (page 12, lines 245-255).

“The difference observed between the central and peripheral VFs was opposite to that predicted by the difference in temporal resolution. The temporal resolution is higher for the peripheral VF than for the central VF [24]; thus, it was predicted that auditory stimuli should be presented earlier for visual stimuli in the peripheral VF than in the central VF condition for one to perceive subjective simultaneity. However, the current observed difference in the PSS was consistent with the difference in visual latency between the central and peripheral VFs. The response time for the visual stimulus was shorter at the central VF than at the peripheral VF [35]. A previous study that manipulae spatial frequency also observed differences in the PSS that were consistent with differences observed in response time [20, 30]. Therefore, in a TOJ task, differences in the PSS between central and peripheral VFs could be attributed to differences in the response time, not temporal resolution.”

Stevenson et al. (2012) reported an opposite TBW difference from the present study. However, their study manipulated a wider range of eccentricities, and a similar PSS difference to the results of Experiment 2 was observed at eccentricities closer to the present study. Therefore, their results are considered to be consistent with the results of this study (page 17, line 376-page 18, line 383).

“Stevenson, Kruger Fister, Barnett, Nidiffer, and Wallace [37] have found a broader TBW in the peripheral VF than in the central VF. However, a higher percentage of simultaneous judgment was observed in the peripheral VF outside of the present experiment (60° and 90° eccentricities) in their study. In the peripheral VF (30° eccentricity), which was closer to the present experiment, the percentage of simultaneous judgment is slightly lower than that in the central VF. Therefore, the TBW of the central VF is assumed to become wider than that of the peripheral VF up to a certain range (at least 30° eccentricity), which is attributed to temporal resolution.”

3. The results of rapid recalibration in SJ and TOJ were in the same direction in the current study, which is inconsistent with Roseboom (2019). On p. 21, if I understand correctly, the author claimed that the effect of rapid recalibration may be cancelled out by the choice-repetition bias in TOJ. However, I do not understand how this explanation is only applied in the periphery rather than in the center, given that the choice-repetition bias should occur in both conditions when they are mixed in a block. In addition, given the fact that response type in TOJ, SJ, and SB are very different from each other, I am also wondering how the choice-repetition bias can be applied in SB but not in SJ.

*Reply:* Previous studies reporting choice-repetition bias in a TOJ task used blurred visual stimuli. Although the present study used a shape-edge visual stimulus, this stimulus was presumably perceived as blurred in the peripheral VF. Thus, a consistent interpretation can be made if the choice-repetition bias occurs only for blurred visual stimuli (page 12, line 256-page 13, line 269).

“The results of normal rapid recalibration for the central VF were inconsistent with the results of previous studies. Roseboom [29] showed that the PSSs shifted in opposite directions from normal rapid temporal recalibration in a TOJ task. Moreover, Keane, Bland, Matthews, Carroll, and Wallis [36] found that opposite-directed PSS shifts were induced by choice-repetition bias in a TOJ task. Choice-repetition bias refers to the tendency to repeat judgments of temporal order of a previous trial on a current trial. Additionally, rapid recalibration was obfuscated by opposite-directed PSS shifts due to a choice-repetition bias [36]. In this study, the choice-repetition bias did not occur only in the central VF. Roseboom [29] and Keane et al. [36] also presented the visual stimulus in the central VF; however, Roseboom [29] used a Gaussian probe and Keane et al. [36] used a small light-emitting diode. Unlike the circle used in this experiment, these visual stimuli had blurred edges. In the present experiment, choice-repetition bias also occurred in the peripheral VF, in which the visual stimuli were perceived as blurred. Therefore, blurred visual stimuli would be necessary for occurring choice-repetition bias in the TOJ task for audio-visual stimuli.”

In Experiment 3, I assumed that the difference in the judged information between the central and peripheral VFs caused the difference in rapid recalibration. Vroomen and Keetels (2020) showed that the PSS of SJ becomes positive, whereas that of SB becomes negative. Although a significant difference was not observed, the PSS of central VF was positive and that of the peripheral VF was negative. Thus, a consistent interpretation can be made if SB perception does not induce rapid recalibration (page 22, line 490-page 23, line 506).

“The PSS values did not significantly differ between the central and peripheral VFs in SB perception. Although there was no significant difference, the signs of PSS values were different between the central and peripheral VFs, a positive value in the central VF and a negative value in the peripheral VF. Vroomen and Keetels [38] showed a discrepancy between the optimal time of sound to induce bounce and maximal audio-visual synchrony in SB displays. The former timing was before the instance of superposition (i.e., negative value), whereas the later timing was after the instance of superposition (i.e., positive value). Thus, in this experiment, participants may have judged the simultaneity between visual events (i.e., contact between two moving visual stimuli) and sound in the central VF, whereas they judged the bounce perception of the two visual stimuli induced by sound in the peripheral VF.

The discrepancy in results between the central and peripheral VF in rapid recalibration could be explained by this difference in judgment. In this experiment, normal rapid recalibration occurred only in the central VF and not in the peripheral VF. The occurrence of rapid recalibration due to SB perception has not been examined. If SB judgment does not cause a change in the PSS due to the timing of sound on a previous trial, it is consistent with results of this experiment. Future studies are needed to confirm this postulation, since there are little experimental data on rapid recalibration using SB perception.”

4. To my knowledge, it is unusual that the rapid recalibration effect is observed in TBW as demonstrated in Experiment 1. Because the rapid recalibration effect in PSS was only observed in the center while the rapid recalibration effect in TBW was observed both in the center and periphery, the explanation on p.11 therefore does not work.

*Reply:* As you have pointed out, the TBW shift during rapid recalibration is unusual. I have also not predicted and interpreted this result. First, few data on TBW have been reported in previous studies of rapid recalibration. Therefore, I would like to elucidate the mechanism underlying this effect in the future. I have added description about this point (page 13, lines 270-278).

“Moreover, the difference in the TBW between audition and vision leads during the rapid recalibration process is a novel finding. In Experiment 1, the TBW width narrowed in the preceding vision leads presentation than in the preceding audition leads presentation. A narrow TBW indicates a high sensitivity to judge the temporal order between visual and auditory stimuli. Therefore, this finding shows that the temporal information of visual precedence in a previous trial increase the sensitivity in a TOJ task for audio-visual stimuli. In previous studies of rapid recalibration (e.g., [18], [29], and [34]), the difference in TBW width between audition and vision leads conditions have not been investigated. Such a difference in the TBW was not predicted in this study, and this needs to be examined in more detail in the future.”

5. I am wondering how the number of participants was determined in each experiment. I can see that the expected number was 20, but only 17 remaining in Experiment 3. I worry that this number is too small (the main effect of modality order was only marginal significant in the rapid recalibration analysis). The SB perception is a subjective experience and therefore a larger individual difference may exist.

*Reply:* The sample size was determined using PANGEA. The result of the calculation showed that the power (1-*β*) was 0.86 with a sample size of 16 participants in each experiment. I also felt that there were many outliers and the sample size became smaller in Experiment 3; however, I did not consider this to have such a large impact.

6. I have some critical questions regarding the experimental designs:
(1) In Experiment 1, the blank display before the visual target was 500-1000 ms, and the other blank after the visual target was 200-710 ms (this information is missing in the main text). It seems that both blanks were occasionally shorter than the largest SOAs between the visual and auditory target (+/- 510 ms).
(2) Technically, there is no +/-0 ms, only 0 ms. Hence, there should be only 9 SOAs in Experiments 2 and 3. Was the number of trials in the 0 ms SOA doubled as compared to other SOAs?
(3) In Experiment 3, how long is the duration of the motion display? A figure to demonstrate the procedure will be great.

*Reply:* (1) I have added a detailed description of the duration of the blank display (page 8, lines 160-167).

“The duration of this blank display was a fixed length of 500 ms, plus an additional SOA when the auditory stimulus was presented prior to the visual stimulus (i.e., 500 ms at the shortest and 1010 ms at the longest). During the target display period, a circle was presented at one of the following three locations: center, left, or right. The tone either preceded or followed the onset of the circle using the SOA that had been drawn randomly from the set. After the target display, a blank display was again presented. The duration of this blank display was a fixed length of 200 ms, plus an additional SOA when the visual stimulus was presented prior to the auditory stimulus (i.e., 200 ms at the shortest and 710 ms at the longest).”

*Reply:* (2) As you have pointed out, the number of trials in the 0 ms SOA doubled compared with other SOAs. I have added descriptions about this point (page 14, lines 305-307).

“One block comprised 80 trials, with 10 trials for each SOA condition (i.e., 16 trials for 0 ms SOA and 8 trials for other SOAs).”

*Reply:* (3) The duration of motion display was 1440 ms in the experiment. I have added descriptions and figure about this point (page 19, lines 417-418).

“Each trial consisted of a 500 ms fixation stimulus followed by motion displays (a duration of 1440 ms, see Fig 6).”

7. More details and clarifications in data analyses are required:
(1) In Experiment 1, please specify how to estimate the PSS and sigma (TBW) based on the cumulative Gaussian function.
(2) In Experiments 2 & 3, please explain the meaning of alpha, PSS and sigma in terms of the Gaussian function, respectively. Were the different alpha scores in the center and periphery critical in Experiment 3?
(3) Did different fitting methods used in Experiment 1 (maximum-likelihood) vs. Experiments 2 & 3 (minimal RMSE) potentially influence the estimation of PSS and sigma?
(4) On p. 19, line 4, were the p value of the two t tests corrected? Were they one- or two-tailed?

*Reply:* (1) I have added the formula for the cumulative Gaussian function in the revised manuscript (page 9, line 184).

$$P \left( response|SOA \right) = \frac{1}{1+e^{\left[ -\frac{1}{sigma}\left( SOA-PSS \right) \right]}}$$

(2) I have added a description of these parameters in terms of the Gaussian function in Experiment 2 (page 15, lines 318-320).

“…these parameters indicated the height, peak position on the SOA axis, and the width of the Gaussian function, respectively.”

The difference in the alpha value indicates the difference in the degree of bounce perception in Experiment 3. However, it is not difficult to determine whether this difference is critical in connection with the results of Experiment 3. I have added a description about this point in the General discussion section (page 26, lines 565-567).

“Moreover, bounce responses differ between the VFs (i.e., the results of a no-sound experiment and alpha values). It is necessary to further investigate whether this difference affects the estimation of the PSS and TBW of SB perception.”

(3) I also calculated the RMSE of fitting in Experiment 1 and these values did not largely differ from the values of Experiments 2 and 3. Therefore, I assume that such a difference in the fitting method would not influence the estimations.

(4) I used the two-tailed one-sample t-tests, and these tests and p values were corrected. I apologize for inserting the incorrect figure regarding these results (Fig 8) in the previous manuscript. I have revised the figure.

8. Based on the results, which paradigm will be recommended for researchers to explore similar issue in the future studies?

*Reply:* I recommend using the explicit paradigm (i.e., TOJ or SJ task), since it was suggested that different judgments occur between VFs in the SB perception based on this study. I have added at description of this point in the revised manuscript (page 27, lines 590-595).

“Furthermore, a paradigm difference in temporal synchrony perception was also observed in this study, among TOJ, SJ, and SB. The present findings suggest that explicit measurements (i.e., TOJ and SJ tasks) are more desirable to examine the temporal synchrony perception of audio-visual stimuli between VFs, because of the different judgments that occur when using an implicit measurement (i.e., SB perception).”

Minor comments:

1. The current title is wordy—it should be condensed.

*Reply:* I have revised the manuscript title per your suggestion.

2. The sound was presented from headphone, so the perceived location was near the participant’s head rather than in the front. Does this influence the results of PSS, TWB, and rapid recalibration since the visual and auditory stimuli were spatially separate (i.e., violating the unity assumption)?

*Reply:* The distance between the participant and the display was approximately 70 cm. Many previous studies investigating the PSS, the TBW, and rapid recalibration have also used headphones to present sound to participants. Therefore, I assume that the use of headphones does not influence these results.

3. Figures 4 & 6: It would be easier to read if the same conditions have the same color code (such as in Figure 2, the center condition has open dots in (a) and white bars in (b,c)). Same suggestion for Figures 3, 5, & 7.

*Reply:* I have revised these figures per your suggestion.

4. A table summarizing all results of three experiments will be helpful.

*Reply:* I have added a table summarizing the results of the experiments per your suggestion.
