# Supplementary material for: Visual field differences in temporal synchrony processing for audio-visual stimuli
Source: PLoS One. 2021 Dec 16;16(12):e0261129. doi: 10.1371/journal.pone.0261129 (PMC8675747; doi:10.1371/journal.pone.0261129)
Supplement: Attachment — Submitted filename: Reply2_PO3.docx [file pone.0261129.s002.docx]

Replies to Reviewer 1’s comments

I appreciate your helpful and valuable comments on this manuscript. I have revised the manuscript to clarify the theoretical motivation and rationale of this study.

*Point 1:* Abstract

The authors mention the three methods they use to measure audio-visual timing performance. No explanation or motivation is provided as to why one might use different measures, or what they actually refer to. The authors correctly describe the main findings associated with each of the various tasks used in this paper. No attempt is offered in the abstract to interpret these task-contingent effects. Why use these different tasks? I would like to see some attempt to understand the variety of effects observed across the different tasks.

*Reply:* I have added the explanation for the use of different measures in this study (lines 22–23), and suggestions from current results (lines 34–36) to the Abstract.

*Point 2:* 39-42

Why do the authors make reference to this asymmetry? It doesn't seem relevant to the point they're making about the potential functional significance of AV synchrony

*Reply:* I have deleted the relevant statements based on your comment.

*Point 3:* 59-60

Of greatest relevance here is that a prolonged period of adaptation is not necessary to observe temporal recalibration.

*Reply:* I have revised the description as per your suggestion (lines 60–61).

*Point 4:* 67

...are composed of at least two spatio-temporal channels.

*Reply:* I have revised the description as per your suggestion (lines 68–69).

*Point 5:* 73-75

This is an over-generalisation. Please tone this back

*Reply:* I have revised the relevant statements to be more precise (lines 74–79).

“The retinal positions of vision have a similar difference in these two channels: The central vision has a low temporal resolution, while the peripheral vision has a high temporal resolution [23]. Furthermore, there is a difference in visual latency between the central and peripheral visual fields (VFs): The response time for the visual stimulus is shorter at the central VF than it is at the peripheral VF [24]. Hence, the processing speed differs between the central and peripheral VFs.”

*Point 6:* 240

Process should be Processes

*Reply:* I have revised the description as per your suggestion (line 252).

*Point 7:* 252

Spelling - manipulate

*Reply:* I apologize for this typo. This has been corrected (line 264).

*Point 8:* 268-269

I don't follow what the authors mean here

Are they saying that blurred edges are a necessary constraint?

*Reply:* Reviewer 2 also could not follow the discussion. I have therefore reinterpreted this result on the basis of information reliability (lines 274–278).

“In this study, choice-repetition bias was suppressed in the central VF. Low information reliability induces a larger choice-repetition bias [39, 40]. Therefore, the reliability of judging temporal order for audio-visual stimuli would be high in the central VF. This speculation needs to be further investigated”

*Point 9:* Table 1 title

Summary of the three experiments' main findings

*Reply:* I have changed the title of Table 1 as per your suggestion.

*Point 10:* 524

Do the authors mean "In our TOJ task"? I don't follow this sentence

*Reply:* I have revised the wording to make it more understandable (line 530-533).

“VF differences in audio-visual temporal synchrony were observed in the TOJ and SJ tasks in this study. In the TOJ task of Experiment 1, the PSS score indicated that the auditory stimulus is presented earlier as a visual stimulus for one to perceive subjective simultaneity in the central than in the peripheral VF condition.”

*Point 11:* 529-530

Can the authors please clarify this statement?

*Reply:* I have clarified this statement (lines 536–539).

“As a preliminary prediction, the VF differences in the PSS and TBW both follow differences in temporal resolution: The PSS score was larger and the TBW width was wider in the central VF than in the peripheral VF.”

*Point 12:* 532

"A TOJ task"

Suggest: TOJ tasks are assumed to be...

*Reply:* I have revised the description as per your suggestion (line 541).

*Point 13:* 535

"a TOJ task"

Suggest: TOJ tasks are proposed

*Reply:* I have revised the description as per your suggestion (line 544).

*Point 14:* 562-563

Please clarify. I don't see how this relates to the previous sentence

*Reply:* I have deleted this sentence as per Reviewer 2’s comment.

*Point 15:* 574-575

Suggest: "Therefore, rapid recalibration may be induced by ...."

*Reply:* I have revised the description as per your suggestion (line 577).

Replies to Reviewer 2’s comments

I appreciate your helpful and valuable comments on this manuscript and have revised it based on the comments.

1. In my previous point 1, I suggested that the author should introduce the critical differences of the mechanisms underlying the three paradigms used here. This is critical to help the author to predict possible results, and to provide readers clearer scope and motivation for the current study.

*Reply:* I have added descriptions regarding the differences in the mechanisms underlying the three paradigms (lines 92–95, lines 103–105).

“The underlying mechanisms differ between the TOJ and SJ tasks. The TOJ task reflects temporal discrimination processes, whereas the SJ task reflects temporal binding processes [26]. Furthermore, the differences between the TOJ and SJ tasks stem from their decisional and response processes [27].”

“Apparent causality among visual and auditory events as SB perception affects audio-visual synchrony perception in early multisensory integration processes [31].”

2. In my previous point 2, I have proposed that the dissociation between processing time and temporal resolution, which may lead to different predictions for PSS and TBW, respectively. Now the author suggested that the visual latency (which is close to the idea of processing time) is shorter in the center than in the periphery, which can explain the PSS result in the TOJ task. There should be hypothesis based on visual latency as a function of visual field addressed in Introduction.

*Reply:* I have added a hypothesis based on visual latency as a function of visual field (lines 74–84).

“The retinal positions of vision have a similar difference in these two channels: The central vision has a low temporal resolution, while the peripheral vision has a high temporal resolution [23]. Furthermore, there is a difference in visual latency between the central and peripheral visual fields (VFs): The response time for the visual stimulus is shorter at the central VF than it is at the peripheral VF [24]. Hence, the processing speed differs between the central and peripheral VFs. The present study confirmed the effects of visual temporal resolution on audio-visual synchrony perception by manipulating the VF in which the visual stimulus is presented (i.e., eccentricity). If synchrony perception for audio-visual stimuli follows differences in temporal resolution, then the TBW of the central VF would be wider than that of the peripheral VF, whereas if it follows the difference in visual latency, then the PSS of the central VF would be lower than that of the peripheral VF.”

3. P. 12, Keane et al. (2020) demonstrated the “choice-repetition bias” account based on reanalyzing Roseboom’s (2019) data; Keane et al., instead, reported a null rapid recalibration effect in Experiment 1, and a typical rapid recalibration effect in Experiment 2. Hence, the author’s “blurry visual information” account cannot work. Following the argument of “choice-repetition bias”, is it possible that the more pronounced rapid recalibration effect in both visual fields was a result of over-estimation due to this bias?

*Reply:* Keane et al. (2020) have shown in Experiment 2 that suppressing the choice-repetition bias leads to the typical rapid recalibration effect in the TOJ task. Therefore, in the TOJ task, the choice-repetition bias would be suppressed in the central VF. The choice-repetition bias is associated with the information reliability. Therefore, I have revised the interpretation on the basis of information reliability (lines 274–278).

“In this study, choice-repetition bias was suppressed in the central VF. Low information reliability induces a larger choice-repetition bias [39, 40]. Therefore, the reliability of judging temporal order for audio-visual stimuli would be high in the central VF. This speculation needs to be further investigated.”

4. Given that the motion display was longer than 1 sec, was the participant’s eye movement monitored or controlled?

*Reply:* The participants were instructed to gaze at the fixation cross during the motion display period. However, eye movement was not monitored. I have added a description of this point (line 430, lines 502–506).

“In the motion display period, participants were instructed to gaze the fixation cross.”

“Audio-visual temporal processing was not affected by VF differences in SB perception. SB perception did not differ in terms of the PSS and TBW between the central and peripheral VFs. However, the duration of motion display was over 1000 ms and participants’ eye movements were not monitored in this experiment. Thus, the participants’ gaze was off the fixation point in the motion display period, which may have affected the current results.”

5. In Experiment 3, given the fact that the PSS was not significantly different in the center and periphery (p = .69), I don’t see any explanation is needed. Specifically, the author’s suggestion that the participants made different judgments in the center and periphery is not convincing, and so is the following paragraph for the rapid recalibration effect.

*Reply:* I have deleted the statements regarding the VF differences in SB perception based on your comment.

6. Table 1: in the summary of rapid recalibration, it is weird to compare the results in the center and periphery either in the A-leading PSS or in the V-leading PSS. The summary should be the comparison between the A-leading PSS and in the V-leading PSS in the center and periphery, respectively.

*Reply:* I have revised Table 1 as per your suggestion.

7. Here are some arguments that I cannot follow:

(1) P. 10, lines 204-205: I do not understand this argument since the results were analyzed and presented separately in the central and peripheral visual field.

(2) P. 25, lines 533-535: If eccentricity effect can be compensated in later visual processing, and the TOJ involved higher-order processing, how could the former explain the PSS difference in the center and periphery in the TOJ?

*Reply:* (1) I did not split the visual field of a previous trial (Trial t-1); the visual field of the current trial (Trial t) was split. I have clarified the description of this point. (lines 216–218).

“The VFs in the previous trial (Trial t-1) were not split because the correspondence of the spatial location between the current (Trial t) and previous (Trial t-1) trials did not affect the rapid recalibration [37].”

(2) Later visual processing compensated the visual field difference of temporal resolution. It is not clear whether the visual field differences in visual latency are compensated in a later visual processing. The visual field difference of the PSS was consistent with that of visual latency in Experiment 1. Therefore, I interpreted that visual latency was not compensated in higher-order visual processing, and that synchrony perception was consistent with the visual field difference of visual latency in the TOJ task.

8. There are still some mistakes in the manuscript:

(1) P. 8, line 170: In Experiment 1, there were 10 SOAs. If there were 80 trials in a block, then there should be only 8 trials for each SOA. Same problem on p. 14, line 306, and p. 19, line 422.

(2) P. 11, line 223, the PSS should be “lower” or “smaller” in the center than in the periphery, since the values were negative.

(3) P. 14, line 298, and p. 19, line 412: it is unclear to me why the author insists to keep the “±0 ms” SOA, since it does not make sense at all.

(4) In the Procedure in Experiment 3, was there also double number of trials at the 0 ms SOA than other SOAs?

(5) P. 28, line 603: “…rapid temporal recalibration occurred only at the periphery VF in the TOJ…” should be at the central VF.

*Reply:* (1) I have revised the descriptions (line 182, 316, and 433).

(2) I have revised the term (line 235).

(3) I have revised the statement as per your suggestion (lines 316–317 and 433–434).

(4) You are right. I have revised the statement (line 308 and 422).

(5) I have revised the description (line 606).
