# Supplementary material for: Visual field differences in temporal synchrony processing for audio-visual stimuli
Source: PLoS One. 2021 Dec 16;16(12):e0261129. doi: 10.1371/journal.pone.0261129 (PMC8675747; doi:10.1371/journal.pone.0261129)
Supplement: Attachment — Submitted filename: Reply3_PO3.docx [file pone.0261129.s003.docx]

Replies to Reviewer 1’s comments

I appreciate your helpful and valuable comments on this manuscript. I have added the discussion related to the inconsistency of results among three methods.

*Main point*

The manuscript is improved and most of my comments have been addressed. It still reads as a collection of disparate results and tasks. Whilst this doesn’t make for a satisfying story it is arguably an accurate reflection of the state of the current empirical literature on AV synchrony perception. For this reason, the paper makes a useful contribution. I encourage the author to make this point in the Discussion.

*Reply:* I have discussed the disparate results among three method in General discussion section (lines 595–601).

“The PSS score tends to be a negative value (i.e., interpreted as synchrony when auditory stimulus leads to the visual stimulus) in a TOJ task compared to an SJ task [28]. SB perception is also likely to show a negative PSS value compared to an SJ task, since causality judgment is performed in SB perception [47]. In this study, the PSS scores indicated a similar tendency to these functional characteristics. One of the potential contributions is that each of these three methods measured different functional characteristics for audio-visual synchrony perception in a series of experiments.”

*Minor point*

Line 81-82

I don't follow the presented logic of why AV temporal bandwidth is predicted to be broader in the central than in the peripheral visual field.

*Reply:* I predicted that temporal bandwidth becomes broader to make the AV synchrony perception tolerant in the central VF, because the timing perception of visual presentation is ambiguous in the central VF due to its low temporal resolution. I have revised the description to clarify this point (lines 80–83).

“If synchrony perception for audio-visual stimuli follows differences in temporal resolution, then the TBW of the central VF would be wider than that of the peripheral VF for tolerant synchrony perception, because the timing perception of visual presentation is ambiguous in the central VF due to low temporal resolution.”

Replies to Reviewer 2’s comments

I appreciate your helpful and valuable comments on this manuscript and have revised it based on the comments. However, I disagree that about separating the three experiments into different papers, because I considered that one of the useful contributions of this paper is to report the inconsistency of results among the three methods.

1-1. The main purpose of the current study, I assume, is stated in the title “visual field differences in temporal processing for audio-visual stimuli”. Given the fact that the author obtained different results in the three experimental paradigms, it is hard for me to judge which result pattern is genuinely attributed to the influence of eccentricity at the level of perceptual processing. Even if the author accepted my previous suggestions that TOJ involves post-perceptual processing compared to SJ, and SB involves causal relations between the audio-visual stimuli, explanations regarding the relationship between the results at difference filed and these mechanisms remains lacking.

*Reply:* I believe that the explanation regarding the VF differences shown in the present study is sufficient. However, the statements were partly unclear in the previous manuscript, and I have, therefore, revised these points.

1-2. For example, in TOJ, the PSS was more negative (i.e., at the auditory-leading side) in the center than in the periphery. On p. 13, the author stated that this difference was consistent with “difference in visual latency between the central and peripheral VFs”. But later the author said “could be attributed to differences in the response time”. Was this a perceptual effect or decisional effect? Would the patter be reversed if the task is changed into “which stimuli comes second”?

*Reply:* I consider that the difference in PSS between the central and peripheral VFs is the perception effect. Breitmeyer (1985) reported that response time reflects differences in visual latency. Thus, the difference in response time between central and peripheral VFs would also be a perceptual effect caused by difference in visual latency. I have added the statements of this point (lines 259–266).

“The difference in the PSS observed between the central and peripheral VFs was consistent with the difference in visual latency between the central and peripheral VFs. The response time for the visual stimulus was shorter in the central VF than in the peripheral VF [24]. Previous studies that manipulated spatial frequency also observed differences in the PSS consistent with the differences observed in response time [19, 29]. The difference in visual latency is reflected in the response time of the visual stimulus [22]. Therefore, in a TOJ task, differences in the PSS between the central and peripheral VFs could be attributed to differences in visual latency rather than temporal resolution.”

1-3. Another example on the top of p. 7: I don’t see the link between “the rapid temporal recalibration in the central and peripheral visual fields” and “the reversed pattern of PSS shift in TOJ and SJ reported by Roseboom”.

*Reply:* There was a mistake in the description; therefore the correct wording has been revised (lines 110–116).

“In addition, the differences in the effects of rapid temporal recalibration between SJ and TOJ tasks were investigated to confirm the effects of VFs on audio-visual temporal processing in this study. Roseboom [32] showed the opposite change in PSS due to rapid temporal recalibration between SJ and TOJ tasks: the PSS changed in the same direction as the stimulus onset asynchrony (SOA) of previous trials in the SJ task and in the opposite direction in the TOJ task. Therefore, this difference in the rapid temporal recalibration between SJ and TOJ tasks was also investigated.”

1-4. One more example is that, on p. 7, if the author’s previous study did not observe different rapid temporal recalibration based on different spatial frequency, then what is the reason to assume that there would be difference in the center vs. peripheral visual fields?

*Reply:* I considered that the VF difference in PSS change between central and peripheral VFs is related to the magnitude of the choice-repetition bias in the TOJ task. In this task, unlike the SJ task, the choice-repetition bias operates the process of PSS change. Thus, it is possible that the magnitude of the choice-repetition bias caused the VF difference in the PSS change, not the rapid recalibration process. I have clarified this explanation in the revised manuscript (lines 267–277).

“The results of normal rapid recalibration for the central VF were inconsistent with the predictions based on previous studies. Roseboom [32] showed that the PSSs shifted in opposite directions from normal rapid temporal recalibration in a TOJ task. Moreover, Keane, Bland, Matthews, Carroll, and Wallis [38] found that opposite-directed PSS shifts were induced by choice-repetition bias in a TOJ task. Choice-repetition bias refers to the tendency to repeat judgments of the temporal order of a previous trial in a current trial. Additionally, rapid recalibration was obfuscated by opposite-directed PSS shifts to a choice-repetition bias [38]. It is possible that the choice-repetition bias was suppressed in the central VF in this study. Low information reliability induces a larger choice-repetition bias [39, 40]. Therefore, the reliability of judging temporal order for audio-visual stimuli would be high in the central VF. This speculation needs to be further investigated.”

2. The author accepted previous suggestions and argued that temporal resolution is related to TBW whereas visual latency is related to PSS (p. 5). However, on p. 13, the authors still contrasted temporal resolution and visual latency as two possible explanations for PSS. The same confusion appeared again on p. 26.

*Reply:* Thank you, I accepted your useful suggestion. However, it is also necessary to explain why visual latency, rather than temporal resolution, affected the PSS score in the TOJ task. I revised the descriptions to avoid confusion and clarify my arguments. (lines 259–266, lines 537–545).

“The difference in the PSS observed between the central and peripheral VFs was consistent with the difference in visual latency between the central and peripheral VFs. The response time for the visual stimulus was shorter in the central VF than in the peripheral VF [24]. Previous studies that manipulated spatial frequency also observed differences in the PSS consistent with the differences observed in response time [19, 29]. The difference in visual latency is reflected in the response time of the visual stimulus [22]. Therefore, in a TOJ task, differences in the PSS between the central and peripheral VFs could be attributed to differences in visual latency rather than temporal resolution.”

“As a preliminary prediction, the VF differences in the PSS and TBW both follow differences in each visual latency and temporal resolution: the PSS score was lower and the TBW width was wider in the central VF than in the peripheral VF. It has been shown that the difference in eccentricity-dependent temporal resolution is observed in the early visual cortex and is compensated later in the cortical visual pathway [42]. TOJ tasks are assumed to be associated with higher-order processing compared to SJ tasks [28, 43]. Therefore, what PSS score was affected by visual latency could be attributed to the visual hierarchy of eccentricity-dependent temporal contrast in a TOJ task.”

3. Some sentences are still hard to follow or incorrect, such as:

(1) p. 4: “Moreover, rapid recalibration, in which a prolonged period of adaptation is not necessary to observe temporal recalibration, has been reported.” – I don’t get it.

(2) p. 8: the sentence regarding the power analysis is too long and many “and”.

(3) p. 15 and p. 20: there were only 9 SOAs in SJ and SB tasks.

(4) p. 19: “The binding for visual and auditory stimuli must be more flexible at the central VF, given the low temporal resolution” I don’t see the relations.

(5) p. 22, line 456: should be Figs 7b and 7c

(6) p. 24 “If SB judgment does not cause a change in the PSS due to the timing of sound in a previous trial” How come a participant’s judgment can cause something?

*Reply:* (1) I revised the description to clarify my intention (lines 58–59).

“Moreover, van der Burg, Alais, and Cass [17] showed temporal recalibration without adaptation period, which they termed “rapid recalibration.””

(2) I revised the sentence as per your comment (lines 138–142).

“To determine the sample size needed for this study, PANGEA (https://jakewestfall.shinyapps.io/pangea/) was used to calculate the power (1-*β*) needed to detect a two-way interaction with following parameters: effect size (*d*) = 0.45, variance of error = 0.333, variance of two-way interaction = 0.083, and the number of condition repetitions = 24. PANGEA indicated a power of 0.86 when a sample size was 16 participants.”

(3) I corrected the statement (line 306 and 420).

(4) I revised description to clarify my argument (lines 383–385).

“Thus, the range of audio-visual synchrony perception would be more tolerant in the central VF due to adapting to low temporal resolution.”

(5) I corrected the description (line 455).

(6) I revised statements to clarify my argument (lines 508–513).

“It is possible that participants performed timing judgment of bounce in the central VF and causal judgment of bounce in the peripheral VF. The occurrence of rapid recalibration due to SB perception has not yet been examined. If participants performed a causal judgment in peripheral VF and the timing information on a previous trial did not cause a change in the PSS in causal judgment, it is consistent with the results of this experiment.”
